# Selection and validation of suitable reference genes for qPCR expression analysis in young *in vitro* grown kohlrabi under cytokinin and sucrose treatments

**DOI:** 10.3389/fpls.2026.1820839

**Published:** 2026-04-29

**Authors:** Anđela Tomić, Jelena Milojević, Martin Raspor, Mariana Stanišić, Branka Uzelac, Slavica Ninković, Tatjana Ćosić

**Affiliations:** Department of Plant Physiology, Institute for Biological Research “Siniša Stanković”—National Institute of the Republic of Serbia, University of Belgrade, Belgrade, Serbia

**Keywords:** carbohydrate metabolism, cytokinins, kohlrabi, qPCR, reference genes, RefFinder, sucrose

## Abstract

Reference genes (RGs) commonly serve as internal controls for normalization of gene expression data in quantitative real-time polymerase chain reaction (qPCR) analyses. To ensure accurate and reliable normalization and interpretation of the results, a systematic validation of RG stability under particular experimental conditions is essential. Our research group previously established a suitable *in vitro* model system for kohlrabi (*Brassica oleracea* var. *gongylodes*) growth and regeneration, encompassing four early developmental stages under varying cytokinin and sugar treatments. As the stability of potential RGs had not yet been evaluated for kohlrabi, we selected and tested suitable RGs for our specific experimental setup. A total of 15 candidate RGs were analyzed using the RefFinder online tool, which integrates four statistical algorithms (geNorm, NormFinder, BestKeeper, and the comparative ΔCt method) to generate a comprehensive stability ranking. *TUA2, ACT7* and *SAND* were identified as the most stable RGs across all experimental subsets, while *GAPB* and *GLUR3.2* exhibited the lowest stability. These findings were validated by qPCR analysis of selected sucrose-related target genes. Our results highlight the importance of context-specific RG validation and provide a valuable resource for gene expression studies in non-model horticultural crops such as kohlrabi.

## 1 Introduction

Reference genes (RGs), often referred to as housekeeping genes, are commonly used as internal controls in quantitative real-time polymerase chain reaction (qPCR) experiments for normalizing gene expression data (Thellin et al., 1999). These genes have critical functions in plant cells and metabolism, and they are assumed to exhibit consistent expression levels across various experimental conditions, tissues, and developmental stages. Nevertheless, different tissues within a plant may have distinct gene expression patterns, and this can impact the stability of RGs (Radonić et al., 2004; Die et al., 2010; Škiljaica et al., 2022). In addition, the suitability of these genes can vary between species and experimental conditions (Tian et al., 2015; Yu et al., 2019; Li et al., 2021; Zeng et al., 2020; Xie et al., 2021; Zhang et al., 2021; Škiljaica et al., 2022; Ferreira et al., 2023).

Examples of commonly used RGs in plants include *actin* (*ACT*), *18S rRNA*, *elongation factor-1α* (*EF-1α*), *tubulin* (*TUB*), and *ubiquitin* (*UBQ*). Genes from the *ACT*, *UBQ* and *TUA* (*tubulin α*) families were once almost exclusively utilized for qPCR normalization due to the presumption of their vital role in cells, until studies showed otherwise — these genes can actually be poor and inappropriate internal controls in certain specific experimental setups (Czechowski et al., 2005; Mizzoti et al., 2018; Knopkiewicz and Wojtaszek, 2019). Moreover, the standard reference gene *18S rRNA*, which generally exhibits stable expression in different species, was proven to be unstable during the development of *Brassica rapa* flower buds (Xu et al., 2014). Furthermore, when the chosen RG displays significant expression variability, it can compromise the normalization process, potentially yielding inaccurate results (Vandesompele et al., 2002).

Therefore, for the selection of appropriate RGs, it is essential to include various tissues and developmental stages of the plant of interest, cultivated in different conditions, such as stress treatments or hormone applications, relevant to the specific experimental setup (Gutierrez et al., 2008; Gao et al., 2012). It is also important to evaluate the stability of RGs over time by analyzing samples collected at different time points during various stages of plant development (Zheng et al., 2018). By systematically assessing the stability of RGs under diverse conditions, more reliable normalization of gene expression data can be ensured in qPCR experiments, leading to more accurate and meaningful results.

Kohlrabi (*Brassica oleracea* var. *gongylodes*) is a nutrient-dense vegetable, providing a good source of vitamins C and K, as well as dietary fiber (Hassan et al., 2011; Ćosić et al., 2023). It also contains minerals like potassium and is low in calories, making it a healthy addition to a balanced diet. The presence of antioxidants, including glucosinolates, contributes to the potential health benefits of this crop (Choi et al., 2010; Paśko et al., 2021). Over the past ten years, our research group has developed a competent protocol for kohlrabi *de novo* shoot organogenesis (DNSO) (Ćosić et al., 2015). The DNSO model system that we currently use generally encompasses several early developmental stages of kohlrabi plants treated with different cytokinins and sucrose concentrations. We have continuously and successfully exploited this system to explore the hormonal homeostasis and the activity of DNSO-related genes in young developing kohlrabi plants (Ćosić et al., 2019; Ćosić et al., 2021; Ćosić et al., 2022). Understanding the molecular basis of DNSO is an active area of research, and new findings may continue to refine our understanding of the intricate processes involved in plant development and regeneration (Ćosić et al., 2023; Pokimica et al., 2024).

In our previous research, we used qPCR analysis to investigate the activity of the genes of interest, as an effectual tool for determining expression profiles. For normalization of detected expression, *actin* was selected as the most commonly applied housekeeping gene (Ćosić et al., 2019; Ćosić et al., 2021). However, the stability of *actin* and other potential RGs has not yet been investigated in kohlrabi. Since our model system includes four different developmental stages of kohlrabi under varying hormone and sugar treatments, it was crucial to validate the stability of RGs for our specific experimental setup. In this context, we used available literature as a key data source for selection of appropriate candidate genes (including Tian et al., 2015; Yu et al., 2019; Zhang et al., 2021; Ferreira et al., 2023).

Based on a review of previously published data in various plant species (Ding et al., 2004; Barsalobres-Cavallari et al., 2009; Wan et al., 2011; Tian et al., 2015; Zeng et al., 2020; Xie et al., 2021; Lin et al., 2022), particularly in the model system *Arabidopsis* (Czechowski et al., 2005; Škiljaica et al., 2022; Ferreira et al., 2023), we selected and assessed the expression stability of fifteen candidate RGs: *Actin-7* (*ACT7*), *SUMO-conjugating enzyme UBC9* (*UBC9*), *NEDD8-conjugating enzyme Ubc12* (*Ubc12*), *tubulin alpha-2 chain* (*TUA2*), *histone H3.3* (*H3.3*), *glutamate receptor 3.2* (*GLUR3.2*), *elongation factor 1-alpha 1* (*eEF1-α1*), *TIP41-like protein* (*TIP41*), *protein SAND-like* (*SAND*), *serine/threonine-protein phosphatase 2A* (*PP2A*)*, polyubiquitin* (*UBQ*), *18S ribosomal RNA* (*18S*), *putative F-box protein At3g16210* (*F-box*), *eukaryotic initiation factor 4A-1* (*eIF4A-1*), and *glyceraldehyde-3-phosphate dehydrogenase* (*GAPB*).

Over the past few decades, numerous efforts have been made to identify the most adequate method to analyze the stability of potential RGs. Today, bioinformatics tools such as geNorm (Vandesompele et al., 2002), NormFinder (Andersen et al., 2004), and BestKeeper (Pfaffl et al., 2004) are widely utilized and can help validate the expression stability of candidate RGs across different tissues and developmental stages. Accordingly, we used RefFinder (Xie et al., 2012; Xie et al., 2023) for the validation of the selected RGs in our kohlrabi model system. This is a highly applicable online software that integrates the three previously listed commonly directed algorithms, along with the comparative ∆Ct method (Silver et al., 2006). These programs use different approaches to rank expression stabilities, and by calculating the geometric mean of the four methods, RefFinder determines the final ranking list.

Accumulation of assimilates in the form of the aboveground stem tuber is a rather distinctive characteristic of kohlrabi (Selman and Kulasegaram, 1967; Ćosić et al., 2020). Since the mechanism of tissue swelling in kohlrabi has been insufficiently explored to date, our ambition is to use our well-optimized DNSO model system to further elucidate the regulation of this process, which is closely interlinked with carbohydrate metabolism and partitioning, especially in the early phases of kohlrabi development. In light of this, three target genes: *sucrose synthase 1* (*SuSy1*), *sucrose-phosphate synthase 1* (*SPS1*), and *cytosolic invertase 1* (*CINV1*), encoding enzymes involved in sucrose metabolism in plants, were used for validation of the selected RGs. The utilization of sucrose by plants requires its cleavage by cytosolic invertase (INV) or by sucrose synthase (SuSy). Invertase facilitates the irreversible hydrolysis of sucrose into glucose and fructose, while SuSy promotes the reversible breakdown of sucrose in the presence of UDP, resulting in fructose and UDP-glucose. Depending on the pH of the medium, SuSy operates in either the synthesis or cleavage direction, predominantly favoring the latter (Schmolzer et al., 2016). In addition, sucrose-phosphate synthase (SPS) is crucial for the production, transport, and storage of sucrose in plants. This enzyme is a soluble, unstable protein present in low quantities, the activity of which is linked to the equilibrium between the accumulation of sucrose and starch in plants (Huang et al., 2017).

Hence, the aim of our work was to analyze the expression stability of a set of 15 candidate reference genes across different stages of early kohlrabi development under elevated sucrose or cytokinin treatments, using available online bioinformatics tools, and relevant target genes related to sucrose metabolism in plants for qPCR validation. We consider our study to be a valuable resource for the scientific community in terms of choosing appropriate reference genes for insufficiently explored plant species and experimental systems that are not commonly used as model systems, such as kohlrabi.

## 2 Materials and methods

### 2.1 Plant material and growth conditions

Commercially available seeds of kohlrabi (*Brassica oleracea* var. *gongylodes*) cv. Vienna Purple (Semenarnacoop, Čačak, Serbia) were surface-sterilized as previously described (Ćosić et al., 2015) by immersion in 70% ethanol for 5 min, followed by 30% commercial bleach (4–6% NaOCl) with a drop of detergent (Fairy; Procter & Gamble, London, UK). After 30 min, the seeds were carefully rinsed using sterile distilled water and aseptically transferred to specific growth media.

Five different growth media were used. Basal, hormone-free growth medium (MS) contained MS mineral salts (Murashige and Skoog, 1962), vitamins (Linsmaier and Skoog, 1965), 3% sucrose, 100 mg L^-1^ *myo*-inositol, and 0.6% agar. MS media containing 6% or 9% sucrose were applied in order to study the effects of high sugar concentration, while MS media supplemented with *trans*-zeatin (*trans*Z) or thidiazuron (TDZ) at 2 mg L^-1^ each, were used for testing exogenous cytokinin (CK) influence. The pH of all media was adjusted to 5.8 before autoclaving at 114 °C and 80 kPa for 25 min. The temperature for the *in vitro* culturing of plants was 25 ± 2 °C and the cool white fluorescent light with an irradiance of 47 µmol m^-2^ s^-1^ was employed providing the 16/8 h light/dark photoperiod.

### 2.2 Sample collection

Following the previously established protocol for *in vitro* growth and *de novo* shoot regeneration, young kohlrabi plants were collected at four different stages of development (T1-T4), as defined in our prior publications (Ćosić et al., 2021; Ćosić et al., 2022): T1 (cotyledon stage), T2 (emergence of true leaves), T3 (callus induction at the stem base), and T4 (differentiation of *de novo* shoots). The stages were defined based on CK-induced morphology. Plants grown on sucrose treatments, along with control plants cultured on basal growth medium, were sampled synchronously with the CK-treated groups. For each developmental stage, three representative plants (excluding regenerated shoots where present) from the same treatment were pooled into a single biological sample. Samples were immediately frozen in liquid nitrogen and then stored at –70 °C until RNA extraction. The complete experimental setup was carried out in three independent biological replicates.

### 2.3 Total RNA extraction and cDNA synthesis

Total RNA of collected kohlrabi samples was extracted using the optimized protocol of Gasic et al. (2004), as previously reported (Ćosić et al., 2019). Briefly, approximately 150 mg of each sample was ground to a fine powder in liquid nitrogen, followed by the addition of CTAB extraction buffer. Two successive extractions with chloroform:isoamyl alcohol (24:1, v/v) were subsequently performed. After overnight precipitation, the final RNA precipitates were resuspended in RNase-free water. Quantity and purity of extracted RNA were assessed by UV absorption spectrophotometry and agarose gel electrophoresis. The procedure included DNase I (Thermo Scientific, Waltham, MA, USA) treatment, after which corresponding cDNA was synthesized using First Strand cDNA Synthesis Kit (Thermo Scientific) from 270 ng of DNase-treated RNA according to the manufacturer’s instructions.

### 2.4 Selection of candidate reference genes and primer design

A list of candidate RGs was compiled from the available literature. Numerous studies regarding the selection of candidate RGs were analyzed to identify commonly used housekeeping genes with stable expression across tissue type, treatment conditions and plant species (Tian et al., 2015; Yu et al., 2019; Li et al., 2021; Xie et al., 2021; Zhang et al., 2021; Škiljaica et al., 2022; Ferreira et al., 2023). Selection was based on candidates belonging to different functional classes. The expression of these genes was then evaluated in young *in vitro*-grown kohlrabi plants across four developmental stages under different sucrose and cytokinin treatments. This approach was used to evaluate the variability and reproducibility of potential housekeeping genes.

The coding sequences of the 15 candidate RGs were acquired from the National Center for Biotechnology Information (NCBI; https://www.ncbi.nlm.nih.gov/). Potential homologs were identified by BLAST searches against the genome and transcriptome sequences of *Brassica oleracea* var. *oleracea* using available data in NCBI. Details of the selected candidate RGs are presented in **Table 1**.

**Table 1:** 15 candidate reference genes and primer sequences used in this study.

Gene abbreviation	Gene name	Accession number	Primer sequence (5‘ to 3’)	Amplicon Size (bp)	PCR Efficiency (E%)	R^2^
*ACT7*	Actin-7	XM_013765231.1	TTCCTCACGCTATCCTCCGT CGTAGTCGAGAGCCACGTAA	159	95.048	0.998
*UBC9*	SUMO-conjugating enzyme UBC9	XM_013745002.1	TCCTGCCCTCACTATCTCCAA TTTTGGGTCCAGGTCCTTGC	150	98.39	0.999
*Ubc12*	NEDD8-conjugating enzyme Ubc12	XM_013773643.1	TGAACCTTCCAAGCTCGTGTA GGATACACAGGAGACACTTGG	142	99.03	0.999
*TUA2*	tubulin alpha-2 chain	XM_013772919.1	TTCGCCCGTGGACATTACA ACCCAAGACCAGACCCAGTT	145	92.40	1
*H3.3*	histone H3.3	XM_013752415.1	ACCGCTCGTAAGTCTACAGG AACAAGCCTCTGGAAAGGGA	198	92.47	0.999
*GLUR3.2*	glutamate receptor 3.2	XM_013749490.1	TGGTGGAGGTTCTGTTACGC GGTTGCTTCCGTTGACCCTA	122	95.84	0.996
*eEF1-α1*	elongation factor 1-alpha 1	XM_013764802.1	GATGACTCCAACCAAGCCCA GATGACACCAACAGCAACCG	106	96.29	0.999
*TIP41*	TIP41-like protein	XM_013735780.1	GTGGGAGAACTGTGAAGAGCA ACCACGAACTTGGCATGACT	155	94.39	0.997
*SAND*	protein SAND-like	XM_013775105.1	TCATCATCCGCCAACGATTTC ATCATCATCGGTGGGGGTTC	141	93.56	1
*PP2A*	serine/threonine-protein phosphatase 2A 65 kDa regulatory subunit A beta isoform	XM_013737765.1	GCTGCTGCCAACAATGTGAA GCAAGAAGAGAAACCGCACG	146	100.22	0.998
*UBQ*	polyubiquitin	XM_013773381.1	ACTCTCACCGGGAAGACCAT TCCCGTCTTCCAACTGCTTT	142	96.97	0.999
*18S*	18S ribosomal RNA	XR_007338012.1	AACGGCTACCACATCCAAGG ACCAGACTTGCCCTCCAATG	163	101.45	0.998
*F-box*	putative F-box protein At3g16210	XM_013729535.1	TGATGGGACGTTATGCGTGA CGGATCGTAACCGAGACCAA	133	99.81	0.998
*eIF4A-1*	eukaryotic initiation factor 4A-1	XM_013736855.1	ACAGTCTCTTCGCTCCGACA CCTGAATCTTCGGTGGGAGAA	122	99.05	0.999
*GAPB*	glyceraldehyde-3-phosphate dehydrogenase GAPB	XM_013749055.1	CGACAACGAGTGGGGTTACA TTCCAAAGGGTCTCCGCTTC	103	94.06	0.998

Forward and reverse primers for all candidate RGs were designed using NCBI Primer-BLAST with the following parameters: melting temperature 60 ± 3 °C, GC percentage of 40–60%, primer lengths of 19–21 bp and product length of 100–200 bp (**Table 1**). Primer properties were further verified using NetPrimer (PREMIER Biosoft International, San Francisco, CA, USA). All primers were synthesized by Microsynth AG (Balgach, Switzerland). Primer specificity in amplifying a single product was confirmed using 1.2% agarose gel electrophoresis after 40 cycles of conventional PCR (**Supplementary Figure 1**). In addition, the specificity of each pair of primers was evaluated by melting curve analysis followed by the amplification in qPCR, as described below.

### 2.5 qPCR analysis

qPCR was performed on a QuantStudio™ 3 Real-Time PCR System (Thermo Fisher Scientific) using a SYBR Green I-based assay (Maxima SYBR Green/ROX Kit, Thermo Scientific). Each 10 μL PCR reaction mixture contained 5 μL of the qRT-PCR Master Mix, 1 μL of cDNA template (corresponding to 13.5 ng cDNA), 2.8 μL of nuclease-free water and 0.6 μL of each primer (forward and reverse, 5 μM). All reactions were run in three technical replicates and each assay included non-template controls (NTCs). Amplification conditions were as follows: initial denaturation at 95 °C for 5 min, followed by 35 cycles of denaturation at 95 °C for 30 s, annealing at 60 °C for 1 min, and extension at 72 °C for 1 min. Associated melting curve analyses were applied by cooling the reactions to 60 °C, followed by increasing the temperature to 95 °C at a ramp rate of 0.1 °C s^-1^, with continuous fluorescence acquisition. The efficiency of PCR reactions was confirmed for each analyzed gene, using 10-fold serial dilutions of specific standards and generating individual standard curves, as previously described (Ćosić et al., 2019). Slope, coefficient of determination (R^2^) and amplification efficiency were automatically calculated using QuantStudio Design and Analysis Software ver. 1.4 (Thermo Fisher Scientific).

### 2.6 Stability evaluation of candidate reference genes

For each qPCR reaction, the threshold-cycle value (Cq) was recorded. Mean Cq values and their variance for the total number of samples (20), as well as for the three main groups (developmental stage, sucrose and cytokinin treatment) were presented using a box plot layout.

The online software RefFinder (https://www.heartcure.com.au/reffinder/; Xie et al., 2012; Xie et al., 2023) was employed to evaluate the expression stability of the selected candidate RGs. This software integrates four commonly used statistical validation algorithms: BestKeeper (Pfaffl et al., 2004), geNorm (Vandesompele et al., 2002), NormFinder (Andersen et al., 2004) and the comparative ∆Ct method (Silver et al., 2006). These algorithms use different approaches to assess and categorize expression stability and have been applied to rank candidate RGs for qPCR experiments across a number of different species.

RefFinder systematically takes ranking information from each algorithm into account and determines the geometric mean of their values for the overall final ranking list (Xie et al., 2012), providing complementary information for the selection of candidate RGs according to their stability (De Spiegelaere et al., 2015; Shakeel et al., 2018).

BestKeeper and the comparative ∆Ct method analyze the stability of candidate RGs using basic Cq values. On the other hand, the relative quantities (RQ) are necessary for geNorm and NormFinder. For each individual replicate in each separate group of data, RQ values were calculated using the 2^−ΔCq^ formula, where ΔCq = (Cq value of each sample) – (the minimum Cq value in each setup), with the prerequisite that the efficiency of qPCR reactions for the distinct sets of primers ranged from the desirable 90% to 105%, as previously established (Livak and Schmittgen, 2001; Ramakers et al., 2003).

For every candidate RG, the correlation coefficient (BestKeeper), M value (geNorm), stability value (NormFinder) and mean SD (comparative ∆Ct method) were determined for the individual sample groups: total (all samples), developmental stage (four developmental stages of kohlrabi, T1-T4, grown on control medium), T1, T2, T3, T4 (kohlrabi grown on all types of media in individual developmental stages), sucrose (four developmental stages of kohlrabi, T1-T4, grown on media supplemented with 3, 6, and 9% of sucrose), cytokinins (four developmental stages of kohlrabi, T1-T4, grown on media supplemented individually with both types of CKs, including control medium), *trans*Z and TDZ (four developmental stages of kohlrabi, T1-T4, grown on media supplemented with *trans*Z or TDZ, respectively).

Using the geNorm algorithm the RG expression stability value (M) was determined, taking into account the average pairwise variation of each gene with all other tested genes. The gene with the lowest M value is considered the most stable (Vandesompele et al., 2002; Jain et al., 2006; Han et al., 2012), with the threshold value set at 0.5 (Hellemans et al., 2007). In addition, the geNorm software (geNorm VBA applet for Microsoft Excel, v. 3.5) performed calculation of pairwise variation (Vn/Vn+1, n represents the number of RGs) to determine the optimal number of RGs required for accurate qPCR data normalization. The cut-off value is 0.15 (Li et al., 2017); Vn/Vn+1 values lower than 0.15 indicate that n is suitable to normalize data and there is no need for including an additional RG (Vandesompele et al., 2002).

NormFinder is also used for calculating the expression stability value, but based on inter- and intra- group variations, where the most stable gene has the lowest stability value (Andersen et al., 2004). BestKeeper assesses the candidate RGs stability based on the standard deviation (SD) and coefficient of variation (CV) of the Cq values in a sample group. Genes with the lowest SD and CV values are the most stable (Pfaffl et al., 2004; Zhu et al., 2013; De Spiegelaere et al., 2015). The comparative ∆Ct method also calculates the average SD to determine the expression stability and ranking of the RGs; the most stable genes exhibit the lowest SD values (Silver et al., 2006).

### 2.7 Validation of reference genes by expression analysis of genes involved in sugar metabolism

The validation of the candidate RGs was performed using three target genes, namely *sucrose synthase 1* (*SuSy1*), *sucrose-phosphate synthase 1* (*SPS1*), and *cytosolic invertase 1* (*CINV1*), encoding enzymes implicated in sucrose metabolism in plants. The expression levels of the target genes were quantified in selected samples, corresponding to developmental stage and T1 stage groups, using the two most stable (*TUA2*, *SAND/eIF4A-1*, *18S*), as well as their geometric means according to geNorm pairwise variation (*TUA2*+*SAND/eIF4A-1+18S*), and the two least stable (*GLUR3.2*, *H3.3*/*GAPB*, *GLUR3.2*) RGs determined according to the comprehensive ranking for each group. The T1 stage was used as a calibrator for relative expression calculations in the developmental stage data set, while the control medium was selected as a calibrator for the T1 set. Additionally, the larger sucrose data set was also analyzed for the purpose of RG validation, with *TUA2*, *PP2A*, and *TUA2+PP2A* selected as the best and *F-box* and *GLUR3.2* as the worst RGs. For each stage, the control medium, containing 3% sucrose, was used as the corresponding calibrator. Primer pairs for the amplification of *SuSy*, *SPS1* and *CINV1* are presented in **Table 2**.

**Table 2:** Primer sequences for the amplification of target genes selected for validation.

Gene abbreviation	Gene name	Accession number	Primer sequence (5‘ to 3’)
*SuSy1*	sucrose synthase 1	XM_013774767.1	GCGTTGTATGAAGCCTTTGGTC CAGCAAGAGTATCAGCAGCC
*SPS1*	sucrose-phosphate synthase 1	XM_013755959.1	ACGGAGATGCTGACTCCTAGA GGCACCATCAACAAACTCAGC
*CINV1*	cytosolic invertase 1	XM_013745260.1	CGTCCTTGGCAACTCCAGAT AGCTCCACCGAGTGTTCTTG

The qPCR reactions were performed as already described above. The relative gene expression levels were calculated using the 2^−ΔΔCt^ method (Livak and Schmittgen, 2001). The data were analyzed with the SAS software (SAS Institute, 2002; SAS/STAT, ver. 9.00; SAS Institute Inc., Cary, NC, USA). One-way ANOVA was used to compare relative expression differences, and the differences found among means were determined using Fisher’s least significant difference (LSD) *post-hoc* test at *p* < 0.05.

## 3 Results

This study evaluates the selection and validation of potential reference genes as internal controls for normalizing expression data. The analysis was conducted within a previously established *in vitro* growth system for kohlrabi, covering four morphological stages (T1–T4) under sucrose or cytokinin treatments. The addition of sucrose or CKs to the culture media modified kohlrabi growth, particularly during the T3 and T4 phases, producing shorter plantlets with thicker stems—most notably under 9% sucrose or TDZ. Despite callus formation occurring in both cases, the capacity for *de novo* shoot development was unique to the cytokinin-treated samples, as described earlier (Ćosić et al., 2021; Ćosić et al., 2022).

### 3.1 Analysis of primer specificity and PCR efficiency

A total of fifteen candidate RGs were chosen for expression stability assessment using RT-qPCR method. The details regarding the candidate gene names, abbreviations, accession numbers, primer sequences, amplicon sizes, amplification efficiencies and correlation coefficients are presented in **Table 1**. Gene expression was assessed at four different developmental stages of young kohlrabi plants cultivated under different cytokinin and sucrose treatments.

For evaluation of the specificity of the designed primers for the selected genes, PCR gel electrophoresis, and melting curve analysis were performed. 1.2% (w/v) agarose gel electrophoresis showed a distinct single band of the expected length for each amplified target fragment for each pair of primers (**Supplementary Figure 1A**). In addition, the occurrence of a single peak in a melting curve was considered evidence of the amplification of a single amplicon, along with the lack of any primer dimer formation (**Supplementary Figure 1B**).

According to the slope of the standard curve, the calculated qPCR efficiency ranged from 92.40% (*TUA2*) to 101.45% (*18S*) for all primer pairs, while R^2^ values extended from 0.996 for *GLUR3.2* to 1.000 for *Ubc12* and *SAND* (**Table 1**). The presented results confirmed that all the 15 primer pairs were successfully designed, complied with the criteria for high reaction specificity and amplification efficiency, and were accordingly appropriate for use in further analysis.

### 3.2 Expression profiles of candidate reference genes in kohlrabi

**Figure 1:**
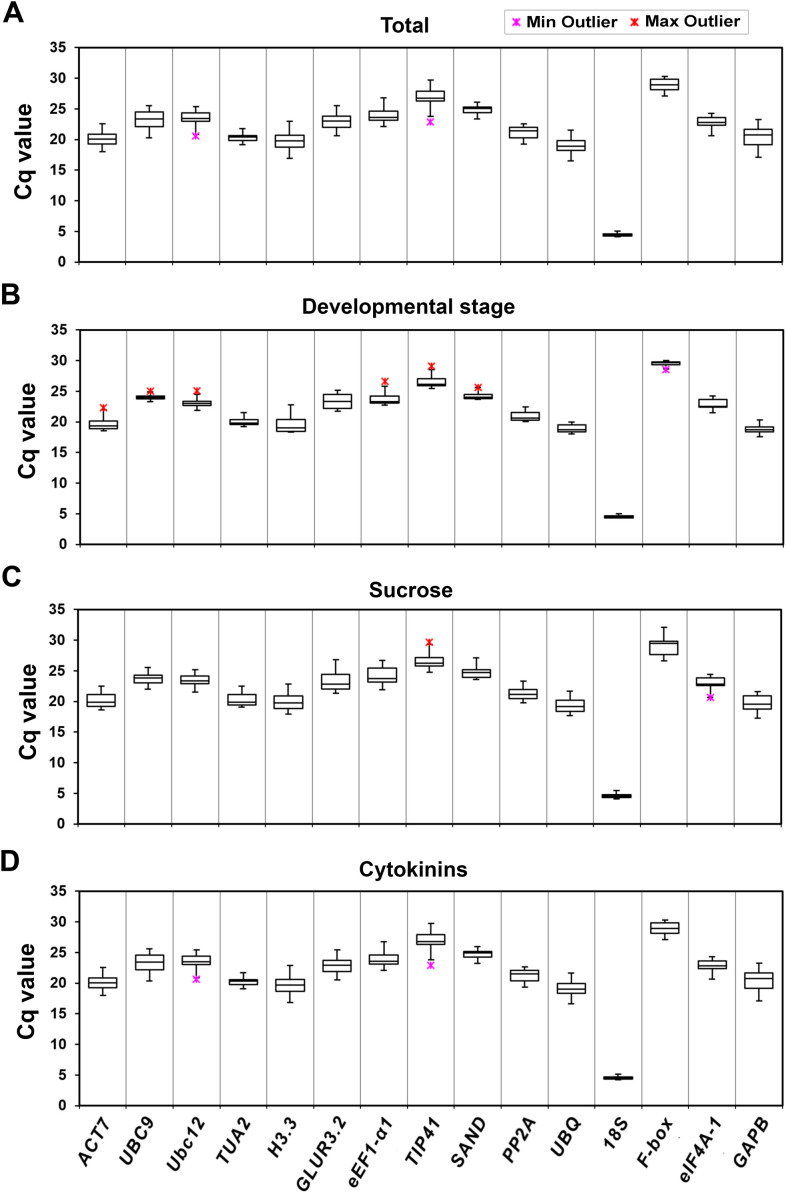
Distribution of qPCR Cq values for the fifteen candidate reference genes in four selected groups of kohlrabi samples: total **(A)**, developmental stage **(B)**, sucrose **(C)** and cytokinin **(D)** treatments. Asterisks denote outliers. The lines across the box indicate median values. The bottom line of each box is determined by the 25^th^ percentile, while the top line is determined by the 75^th^ percentile. The bottom and top whiskers are defined by the 5^th^ and 95^th^ percentiles, respectively.

Cq values of fifteen candidate RGs in different kohlrabi samples, obtained by qPCR data analysis, are presented as boxplots in **Figure 1**. Four main groups of collected samples were selected for the presentation of average Cq values: total, developmental stage, sucrose and cytokinin treatments. Bearing in mind that a low Cq value in the qPCR method for gene activity quantification indicates a high gene expression level (and vice versa), *18S* was shown to be the gene with the highest expression, while *F-box* had the lowest expression in all four individual groups.

Average Cq values varied from 4.55 (for *18S* under CK treatment) to 29.50 (for *F-box* in the developmental stage group), indicating significant variability among the analyzed candidate genes. Furthermore, the *18S* expression levels were shown to be the least variable (ΔCq = 0.89; the minimum and maximum Cq values were 4.27 and 5.16, respectively) among all samples, demonstrating greater stability compared to the others in each tested condition/stage. This was followed by *TUA2* (ΔCq = 2.23), and *eIF4A-1* (ΔCq = 2.51), whereas the *TIP41* expression levels showed the highest variability (ΔCq = 4.85, ranging from 23.98 to 28.83) (**Figure 1**). Since the variability of the other RGs tested was fairly similar, further analysis of these genes was needed.

### 3.3 Expression stability evaluation of the candidate reference genes

Evaluation of the expression stability of the selected fifteen candidate RGs was performed using RefFinder software. By encompassing four individual statistical algorithms (geNorm, NormFinder, BestKeeper and ∆Ct), RefFinder determines the overall final ranking list of the selected candidate genes according to their expression stability. The analysis was performed for the total group of young kohlrabi samples and for the individual experimental sets, including the developmental stage, T1, T2, T3 and T4 stages, sucrose and cytokinin treatments, as well as the individual *trans*Z and TDZ treatments.

#### 3.3.1 geNorm

**Figure 2:**
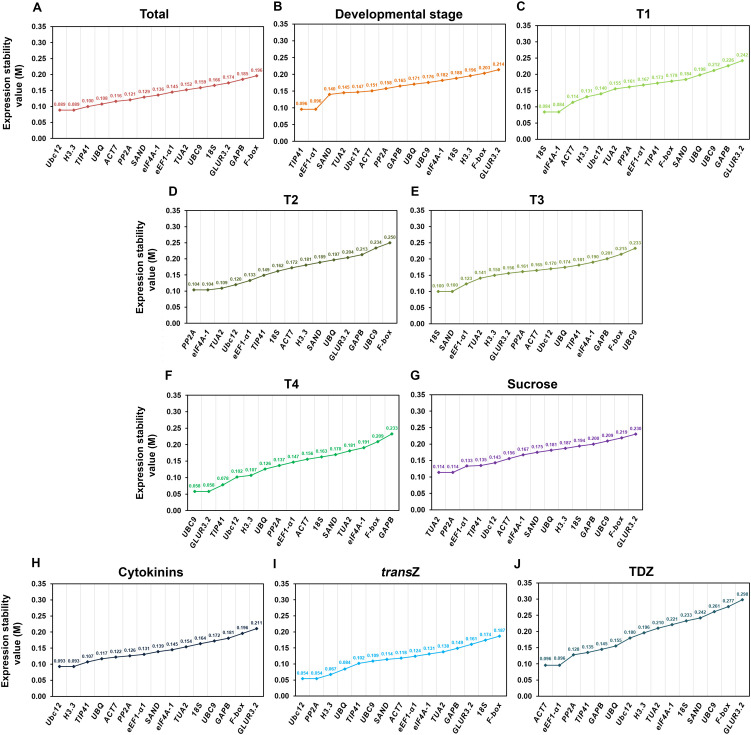
Average expression stability values **(M)** of the fifteen candidate reference genes in young kohlrabi plants, calculated by geNorm within the RefFinder software across different experimental sets. **(A)** Total (all samples), **(B)** developmental stage, **(C–F)** individual developmental stages (T1—T4), **(G)** sucrose treatment, **(H)** cytokinin treatment, **(I)** *trans*Z treatment, and **(J)** TDZ treatment. Genes are ranked in descending order of stability (from left to right). *trans*Z = *trans*-zeatin; TDZ = thidiazuron.

The results of the geNorm analysis showed that the M values of all evaluated candidate RGs across all experimental sets were lower than the 0.5 threshold value, suggesting that these genes demonstrate satisfactory stability and are hence suitable for use as RGs (**Figure 2**).

The geNorm software version used in the present work provided a stability ranking list in which two genes shared the position of the most stable gene. For the total sample set, *Ubc12* and *H3.3* were the two most stable genes, with an M value of 0.089 (**Figure 2A**), while *TIP41* and *eEF1-α1* (M = 0.096) were the most stable genes for the developmental group (**Figure 2B**). Regarding individual developmental stages, *18S* and *eIF4A-1* (0.084), *PP2A* and *eIF4A-1* (0.104), *18S* and *SAND* (0.1), and *UBC9* and *GLUR3.2* were identified as the most stable in T1, T2, T3 and T4, respectively (**Figures 2C–F**). Furthermore, *TUA2* and *PP2A* were identified as the two most stable RGs during sucrose treatment, with an M value of 0.114 (**Figure 2G**). *Ubc12* and *H3.3* were once again the RGs with the lowest M value (0.093) for cytokinin-treated samples (**Figure 2H**), while *Ubc12* and *PP2A* (0.054), and *ACT7* and *eEF1-α1* (0.096) were calculated as the most stable for the *trans*Z and TDZ subsets, respectively (**Figures 2I, J**). Conversely, in almost all experimental groups, *GLUR3.2*, *F-box* and *GAPB* were found to be among the least stable genes, with the exception of the T4 developmental stage (**Figure 2F**).

**Figure 3:**
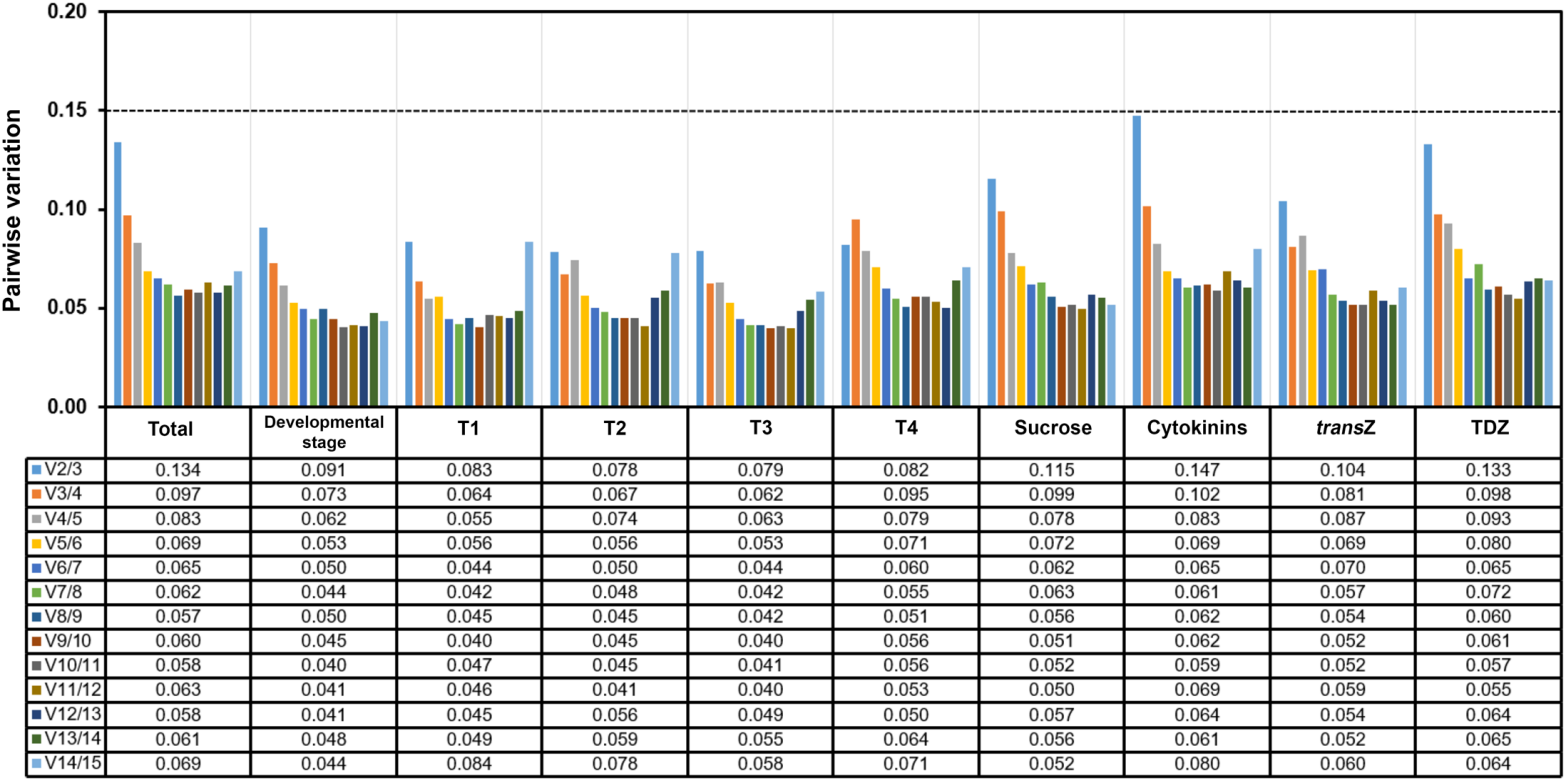
Pairwise variation (Vn/Vn+1) analysis between normalization factors NFn and NFn+1 (n = number of reference genes), performed for the fifteen candidate reference genes using the geNorm program across all kohlrabi samples (total) and sample groups (developmental stage, individual developmental stages (T1–T4), sucrose, cytokinin, and individual *trans*Z and TDZ treatments). The dashed horizontal line represents the cut-off value of 0.15. *trans*Z = *trans*-zeatin; TDZ = thidiazuron.

In addition, geNorm software was used for pairwise variation analysis (Vn/Vn+1, where n represents the number of RGs) in order to determine the optimal number of RGs required for accurate RT-qPCR normalization. Gene groups with Vn/Vn+1 value below 0.15 are considered sufficient for data normalization. According to our analysis, the normalization factors calculated for the first two (NF2) and three (NF3) genes in the geNorm ranking, presented as V_2_/V_3_ values, were lower than the established cut-off value of 0.15 in all experimental sets (**Figure 3**, horizontal line). These results imply that the use of the geometric mean of the two top-ranked RGs would be adequate for data normalization, as would the geometric mean of three or more genes.

#### 3.3.2 NormFinder

**Figure 4:**
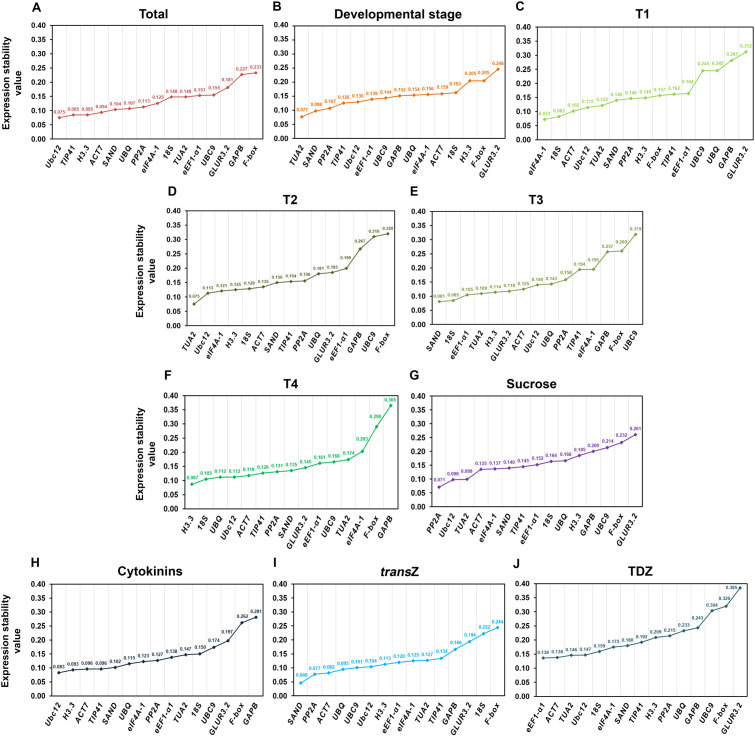
Expression stability values of the fifteen candidate reference genes in young kohlrabi plants, calculated by NormFinder within the RefFinder software across different experimental sets. **(A)** Total (all samples), **(B)** developmental stage, **(C–F)** individual developmental stages (T1–T4), **(G)** sucrose treatment, **(H)** cytokinin treatment, **(I)** *trans*Z treatment, and **(J)** TDZ treatment. Genes are ranked in descending order of stability (from left to right). *trans*Z = *trans*-zeatin; TDZ = thidiazuron.

According to NormFinder, the most stable genes in our case were identified as follows: *Ubc12* and *TIP41* for the total samples (**Figure 4A**), *TUA2* and *SAND* for the developmental stage (**Figure 4B**), *eIF4A-1* and *18S* for T1, *TUA2* and *Ubc12* for T2, *SAND* and *18S* for T3, and *H3.3* and *18S* for the T4 stage (**Figures 4C–F**). *PP2A* and *Ubc12* were the top two most stable genes under sucrose treatment (**Figure 4G**), while *Ubc12* and *H3.3* were identified as the most stable for the overall cytokinin treatment (**Figure 4H**). For the individual *trans*Z and TDZ treatments, *SAND* and *PP2A*, and *eEF1-α1* and *ACT7* showed high expression stability, respectively (**Figures 4I, J**). The expression stability values of the listed highly stable RGs in the experimental groups ranged from 0.046 to 0.138.

The RGs recommended by NormFinder were not highly consistent with those obtained by geNorm analysis. However, despite the differences in rankings between these two approaches, the most stable genes were essentially the same across the various experimental sets presented (**Figure 4**). In addition, *F-box*, *GLUR3.2*, and *GAPB* were the least stable genes in all samples/treatments, which is also consistent with the geNorm output.

#### 3.3.3 BestKeeper

**Figure 5:**
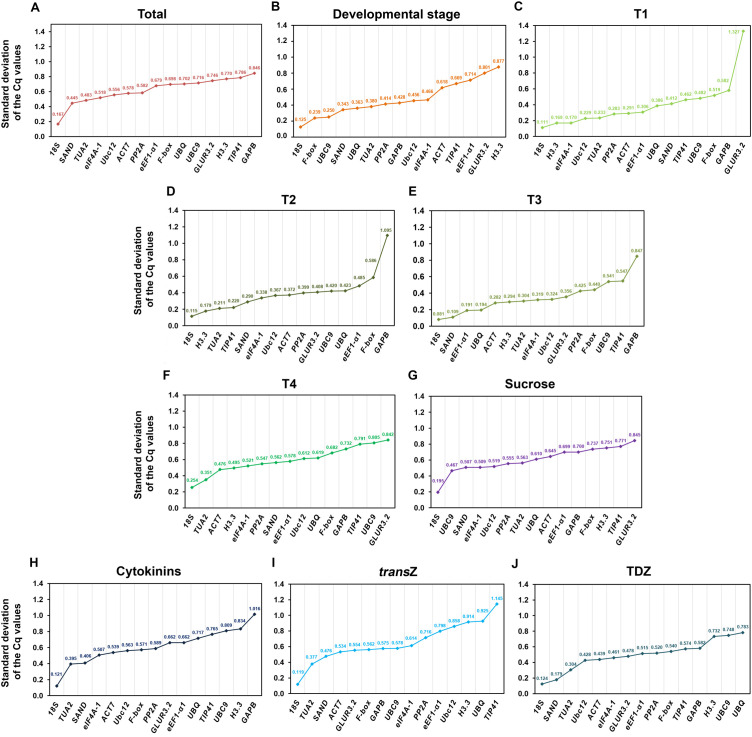
Standard deviation (SD) of Cq values of the fifteen candidate reference genes in young kohlrabi plants, calculated using BestKeeper within the RefFinder software across different experimental sets. **(A)** Total (all samples), **(B)** developmental stage, **(C–F)** individual developmental stages (T1–T4), **(G)** sucrose treatment, **(H)** cytokinin treatment, **(I)** *trans*Z treatment, and **(J)** TDZ treatment. Genes are ranked in descending order of stability (from left to right). *trans*Z = *trans*-zeatin; TDZ = thidiazuron.

*18S* was uniquely shown to be the most stable of the fifteen analyzed RGs across all tested experimental sets according to BestKeeper. SD values for *18S* ranged from 0.081 to 0.254 (**Figure 5**).

As for the least optimal RGs, the program identified several candidates, particularly *GAPB* and *GLUR3.2.* For the total samples (**Figure 5A**), T2 and T3 developmental stages (**Figures 5D, E**), and the cytokinin treatment (**Figure 5H**), *GAPB* exhibited the highest SD values (0.846, 1.095, 0.847, and 1.016, respectively), while *GLUR3.2* had the lowest stability for T1 and T4 developmental stages (**Figures 5C, F**), as well as for the treatment with elevated sucrose (**Figure 5G**), with SD values of 1.327, 0.842 and 0.845. *H3.3* (0.877) was the least stable gene for the general developmental stage (**Figure 5B**), while *TIP41* (1.145) and *UBQ* (0.783) were the least stable for the individual *trans*Z and TDZ subsets, respectively (**Figures 5I, J**).

#### 3.3.4 ∆Ct method

**Figure 6:**
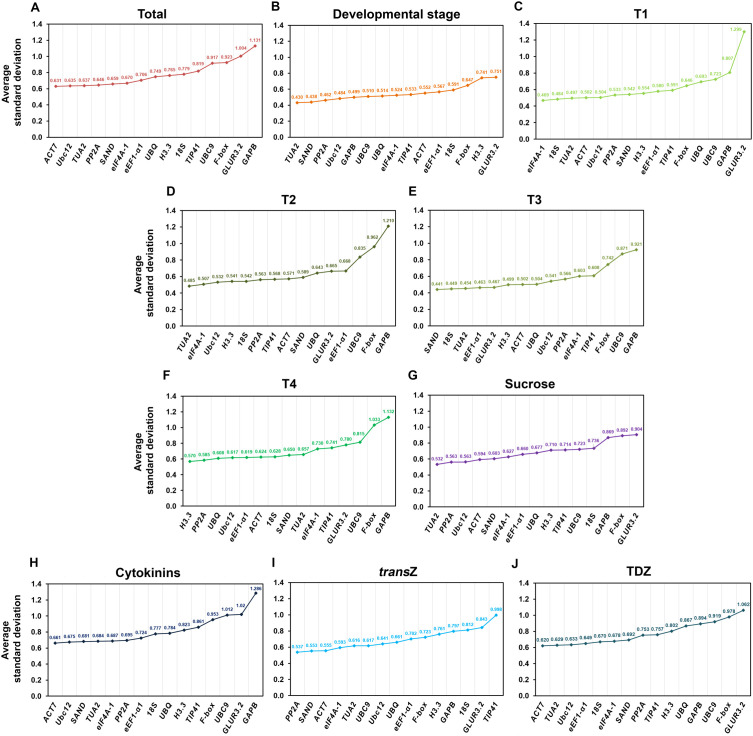
Average standard deviation (SD) of Cq values of the fifteen candidate reference genes in young kohlrabi plants, calculated by the comparative ∆Ct method within the RefFinder software in different experimental sets. **(A)** Total (all samples), **(B)** developmental stage, **(C–F)** individual developmental stages (T1–T4), **(G)** sucrose treatment, **(H)** cytokinin treatment, **(I)** *trans*Z treatment, and **(J)** TDZ treatment. Genes are ranked in descending order of stability (from left to right). *trans*Z = *trans*-zeatin; TDZ = thidiazuron.

As for the comparative ∆Ct method, a RG with the most stable expression is the one with a lower SD, as well (**Figure 6**). *ACT7* was the top-ranked gene for the total samples (0.631), cytokinins (0.661) and individual TDZ treatment (0.620) (**Figures 6A, H, J**). Regarding the general developmental stage, T2 and sucrose treatment, *TUA2* was ranked as the most stable gene with the following SD values: 0.430, 0.485, and 0.532, respectively (**Figures 6D, G, J**). In contrast, *eIF4A-1* (0.469; **Figure 6C**)*, SAND* (0.441; **Figure 6E**) and *H3.3* (0.570; **Figure 6F**) exhibited the highest stability for the T1, T3 and T4 stages, respectively. *PP2A* showed the lowest SD value (0.537) for TDZ treatment (**Figure 6I**).

Consistently, *GAPB* and *GLUR3.2* exhibited significant ∆Ct deviation throughout the experimental sets, implying lower expression stability of these genes in the studied tissues (**Figure 6**).

#### 3.3.5 Comprehensive ranking of the candidate reference genes

In order to provide a comprehensive assessment of the fifteen candidate RGs in varying conditions and developmental stages of young kohlrabi, further analysis was performed via the RefFinder software by calculating a comprehensive ranking based on the geometric mean of four major algorithms.

**Figure 7:**
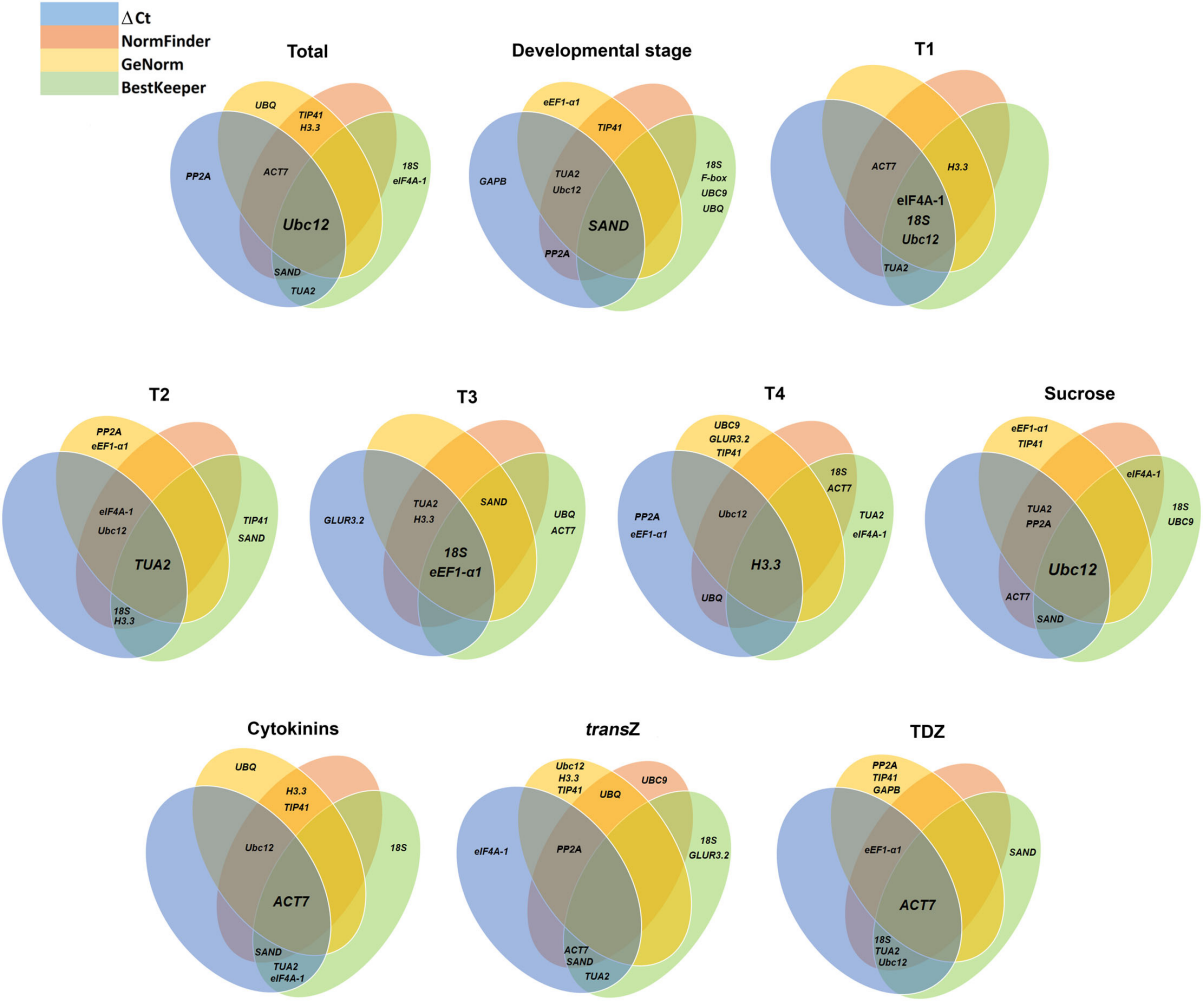
Venn diagram showing the top five most stable reference genes identified by ∆Ct (blue), NormFinder (orange), geNorm (yellow) and BestKeeper (green). Overlapping regions highlight genes consistently ranked as stable across multiple methods. *trans*Z = *trans*-zeatin; TDZ = thidiazuron.

The comprehensive ranking results are shown in **Table 3**. In this analysis, the most stable candidate RGs for total samples were *ACT7* and *TUA2*, and the same results were detected for TDZ treatment. *TUA2* and *SAND* were the most stable RGs in different developmental stages. As for individual stages, *eIF4A-1* and *18S*, *TUA2* and *eIF4A-1*, *SAND* and *18S*, and *H3.3* and *PP2A* exhibited the highest stability in T1, T2, T3 and T4, respectively. Under the sucrose treatment, *TUA2* and *PP2A* were the best choices, while *ACT7* and *Ubc12* were top-ranked genes when cytokinins were applied. Finally, *PP2A* and *SAND* were the two most stable RGs in *trans*Z treatment. These rankings of stable RGs selected under various experimental conditions are slightly different from the five most stable genes selected by the four algorithms separately as shown in **Figure 7**.

**Table 3:** Comprehensive assessment ranking of the expression stability for the 15 candidate reference genes, as computed by RefFinder.

Ranking	Total	Developmental stage	T1	T2	T3	T4	Sucrose	Cytokinins	*trans*Z	TDZ
1	*ACT7* (2.06)	*TUA2* (1.57)	*eIF4A-1* (1.32)	*TUA2* (1.32)	*SAND* (1.19)	*H3.3* (2.11)	*TUA2* (1.63)	*ACT7* (1.50)	*PP2A* (1.78)	*ACT7* (1.97)
2	*TUA2* (2.06)	*SAND* (2.63)	*18S* (1.68)	*eIF4A-1* (2.21)	*18S* (1.41)	*PP2A* (2.21)	*PP2A* (2.45)	*Ubc12* (2.45)	*SAND* (2.71)	*TUA2* (2.34)
3	*PP2A* (3.25)	*PP2A* (2.82)	*TUA2* (4.05)	*18S* (3.31)	*eEF1-α1* (3.46)	*UBQ* (3.31)	*Ubc12* (3.08)	*SAND* (2.71)	*ACT7* (3.13)	*Ubc12* (2.91)
4	*Ubc12* (3.31)	*UBC9* (5.24)	*ACT7* (4.09)	*Ubc12* (3.98)	*TUA2* (4.21)	*18S* (3.98)	*SAND* (4.40)	*TUA2* (3.56)	*eIF4A-1* (3.46)	*18S* (3.31)
5	*SAND* (3.98)	*GAPB* (5.32)	*H3.3* (4.43)	*H3.3* (4.09)	*UBQ* (5.66)	*ACT7* (4.36)	*ACT7* (4.90)	*eIF4A-1* (4.47)	*TUA2* (4.53)	*eEF1-α1* (3.56)
6	*eIF4A-1* (5.42)	*Ubc12* (5.42)	*Ubc12* (4.47)	*PP2A* (5.58)	*GLUR3.2* (6.22)	*eEF1-α1* (5.38)	*eIF4A-1* (5.42)	*18S* (5.03)	*UBC9* (6.62)	*SAND* (5.12)
7	*18S* (5.61)	*UBQ* (6.12)	*PP2A* (6.48)	*TIP41* (5.79)	*ACT7* (6.4)	*Ubc12* (5.42)	*18S* (6.45)	*PP2A* (6.45)	*18S* (6.85)	*eIF4A-1* (5.42)
8	*eEF1-α1* (7.24)	*18S* (6.45)	*SAND* (8.05)	*ACT7* (7.74)	*H3.3* (6.48)	*TUA2* (5.80)	*UBC9* (7.01)	*eEF1-α1* (7.65)	*Ubc12* (7.36)	*PP2A* (8.49) )
9	*UBQ* (8.46)	*F-box* (8.14)	*eEF1-α1* (8.71)	*SAND* (7.77)	*Ubc12* (9.00)	*SAND* (7.97)	*UBQ* (7.74)	*UBQ* (9.19)	*UBQ* (8.56)	*TIP41* (9.19)
10	*H3.3* (10.13)	*eIF4A-1* (8.18)	*TIP41* (9.72)	*UBQ* (10.72)	*eIF4A-1* (10.16)	*eIF4A-1* (8.41)	*eEF1-α1* (7.91)	*F-box* (10.49)	*F-box* (8.80)	*H3.3* (10.68)
11	*TIP41* (11.41)	*TIP41* (9.93)	*UBQ* (11.39)	*GLUR3.2* (10.98)	*PP2A* (10.24)	*TIP41* (11.47)	*H3.3* (9.87)	*H3.3* (10.59)	*eEF1-α1* (9.46)	*UBQ* (11.89)
12	*UBC9* (11.74)	*ACT7* (9.97)	*F-box* (11.47)	*eEF1-α1* (11.70)	*TIP41* (12.47)	*GLUR3.2* (12.69)	*TIP41* (11.14)	*TIP41* 1(1.24)	*GAPB* (10.49)	*GAPB* (12.00)
13	*F-box* (11.86)	*eEF1-α1* (11.47)	*UBC9* (12.49)	*UBC9* (12.47)	*F-box* (12.74)	*F-box* (13.18)	*GAPB* (12.47)	*GLUR3.2* (12.31)	*GLUR3.2* (10.82)	*GLUR3.2* (12.4)
14	*GLUR3.2* (13.47)	*GLUR3.2* (14.49)	*GAPB* (14.00)	*F-box* (14.00)	*UBC9* (13.74)	*UBC9* (13.24)	*F-box* (13.47)	*UBC9* (13.24)	*H3.3* (11.47)	*F-box* (12.87)
15	*GAPB* (15.00)	*H3.3* (14.49)	*GLUR3.2* (15.00)	*GAPB* (15.00)	*GAPB* (15.00)	*GAPB* (14.19)	*GLUR3.2* (15.00)	*GAPB* (15.00)	*TIP41* (15.00)	*UBC9* (13.24)

In addition, the comprehensive assessment showed that in most cases, *GAPB* and *GLUR3.2* were generally the least stable RGs (**Table 3**). This is in accordance with the rankings obtained using individual algorithms.

### 3.4 Validation of selected reference genes by RT−qPCR

**Figure 8:**
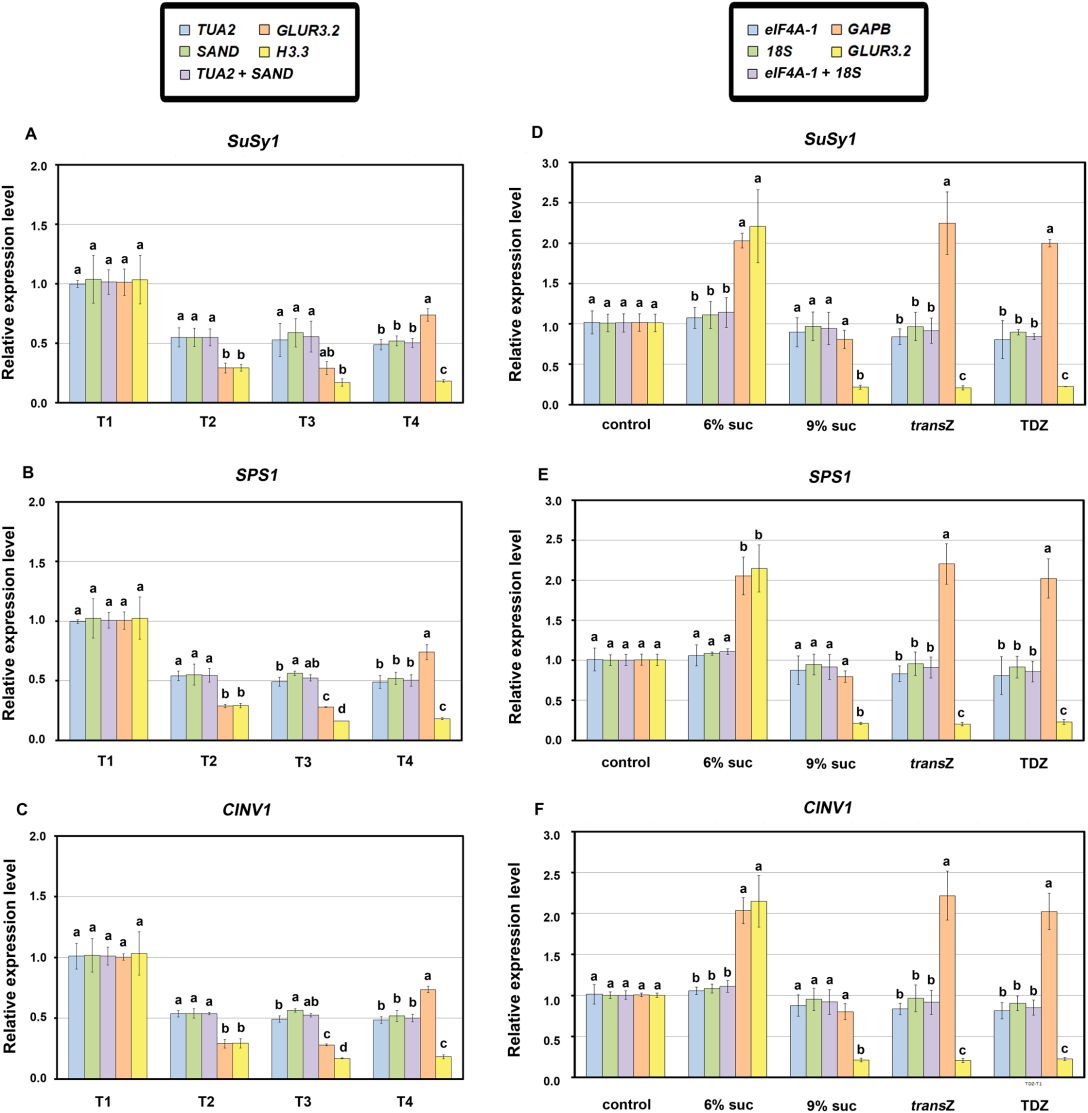
Relative expression levels of *SuSy1*, *SPS1* and *CINV1* coding for sucrose synthase 1, sucrose-phosphate synthase 1 and cytosolic invertase 1, respectively, involved in carbohydrate metabolism, normalized to different reference genes during four stages of early kohlrabi development **(A–C)** and in response to sucrose and cytokinin treatments at the T1 stage **(D-F)**. The expression levels of the target genes were quantified in selected samples using the two most stable (*TUA2*, *SAND, TUA2+SAND / eIF4A-1*, *18S, eIF4A-1+18S*) and the two least stable (*GLUR3.2*, *H3.3* / *GAPB*, *GLUR3.2*) reference genes determined according to the comprehensive ranking for each group. Data represent means ± SE, where different letters denote significant differences according to the Fisher’s least significant difference (LSD) *post-hoc* test (*p* < 0.05) within each developmental stage or treatment. *trans*Z = *trans*-zeatin; TDZ = thidiazuron.

To confirm the expression stability of the selected RGs, three genes involved in sucrose metabolism were chosen as targets for RT-qPCR analysis: *SuSy1* (*sucrose synthase 1*), *SPS1* (*sucrose-phosphate synthase 1*) and *CINV1* (*cytosolic invertase 1*). We selected the two most stable and the two least stable RGs according to the comprehensive ranking results for each experimental group analyzed, and used these RGs for normalization of gene expression data. For the developmental stage experimental set tested on control medium, *TUA2* and *SAND* were used as the best, and *GLUR3.2* and *H3.3* as the worst RGs (**Figures 8A–C**). Conversely, *eIF4A-1* and *18S*, along with *GAPB* and *GLUR3.2*, were used as the most and the least stable genes, respectively, in response to sucrose and cytokinin treatments in the T1 developmental stage (**Figures 8D–F**). According to the pairwise variation results, which indicated that the geometric mean of the two top-ranked RGs would be sufficient for data normalization, the combinations *TUA2*+*SAND* (**Figures 8A–C**) and *eIF4A-1+18S* (**Figures 8D–F**) were applied as well.

The analysis revealed that when the two most stable genes (*TUA2* and *SAND*) and their geometric mean were employed for normalization of *SuSy1*, *SPS1* and *CINV1* in the developmental stage set, statistically significant differences were detected in the expression patterns of all subsets when compared to the normalization using the two least stable RGs (*GLUR3.2* and *H3.3*) (**Figures 8A–C**, respectively). The exception was the T1 stage, which was used as a calibrator for relative expression calculation. Moreover, in most cases, there were no significant differences in the relative expression of all target genes when normalized using the most stable RGs across all subsets. A similar situation was observed in the T1 experimental set (**Figures 8D–F**), where using *eIF4A-1* and *18S* (the two most stable RGs) for normalization resulted in target gene expression levels that statistically differed from those normalized with *GAPB* and *GLUR3.2*. However, the 9% sucrose treatment was an exception, since normalization using *GAPB* showed no statistically significant differences compared to the use of the two most stable RGs, or their combination, for all three target genes (**Figures 8D–F**).

When the expression normalization using the two most stable RGs was considered in developmental data set, all three target genes involved in sugar metabolism showed varying degrees of downregulation in comparison to the calibrator, i.e. T1 stage (**Figures 8A–C**). As for T1 set, the changes in expression levels of target genes on sucrose and CK treatments were minimal compared to control medium when stable RGs were applied for data standardization (**Figures 8D–F**). On the other hand, normalization with low-stability RGs, particularly *GAPB*, showed distinctly different results with significantly increased activity of all target genes under 6% sucrose and CK treatments in the T1 set (**Figures 8D–F**). These findings indicate that the use of reliable RGs plays an important role in the accurate standardization of target genes during early kohlrabi development.

**Figure 9:**
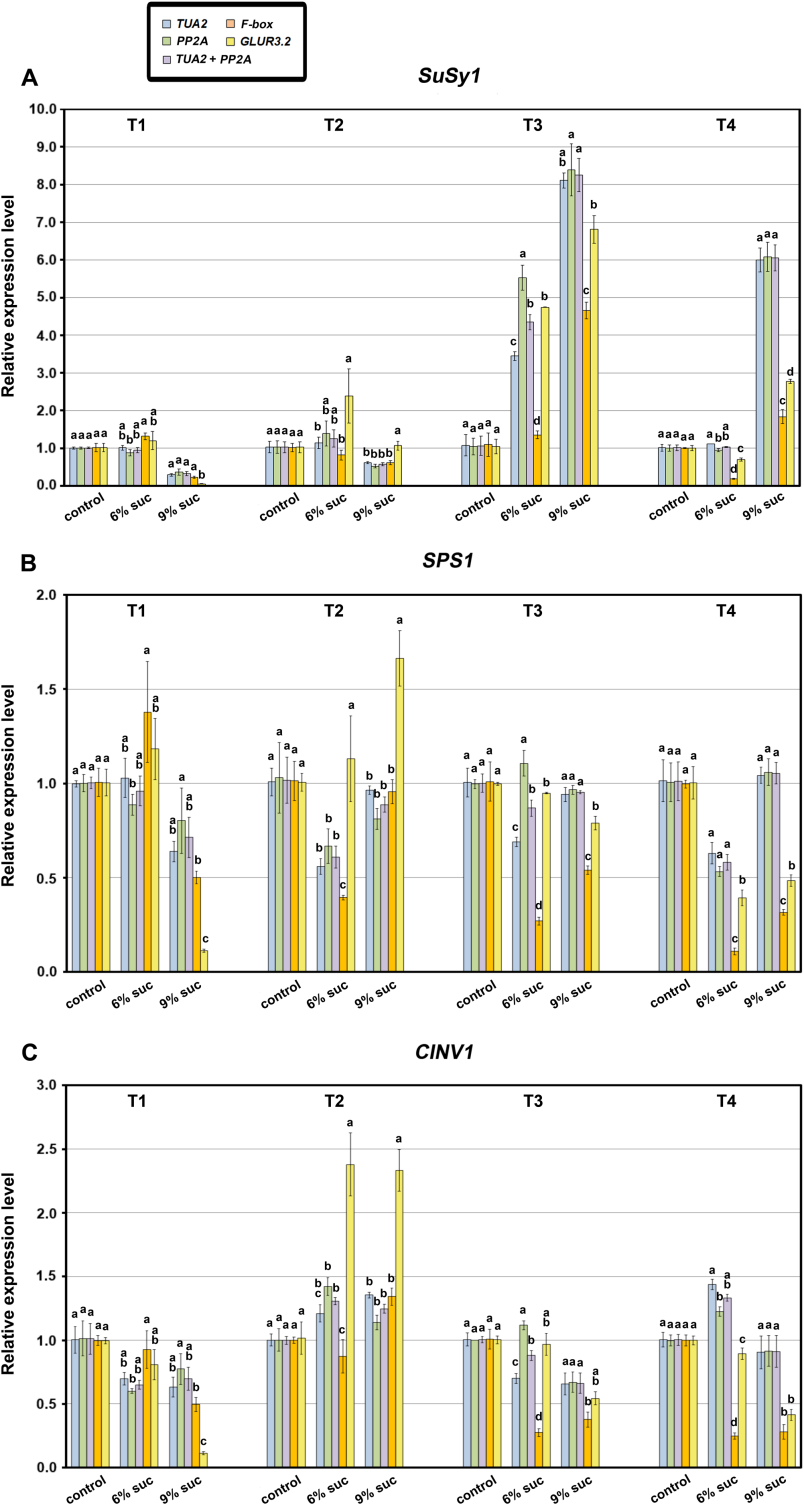
Relative expression of *SuSy1* **(A)**, *SPS1* **(B)** and *CINV1* **(C)** coding for sucrose synthase 1, sucrose-phosphate synthase 1 and cytosolic invertase 1, respectively, involved in carbohydrate metabolism, normalized by different reference genes in response to sucrose treatments across four selected kohlrabi developmental stages (T1–T4). The expression levels of the target genes were quantified in selected samples using the two most stable (*TUA2*, *PP2A, TUA2+PP2A*) and the two least stable (*F-box*, *GLUR3.2*) reference genes determined according to the comprehensive ranking for each group. Data represent mean ± SE, where different letters denote significant differences according to the Fisher’s least significant difference (LSD) *post-hoc* test (*p* < 0.05) among treatments within each developmental stage. *trans*Z = *trans*-zeatin; TDZ = thidiazuron.

Furthermore, a similar pattern was observed for the tested carbohydrate-related genes when the larger sucrose dataset was analyzed. In this case, *TUA2*, *PP2A*, and their combination (*TUA2+PP2A*) were used as the most stable RGs, while *F-box* and *GLUR3.2* were the least stable alternatives (**Figure 9**). In the majority of cases, there was no statistically significant difference in the relative expression values of all target genes when normalized using the most stable RGs across all subsets. However, with the more complex dataset, the more exceptions could be observed, such as 6% sucrose treatment, especially in the T3 developmental stage. More importantly, significant differences were recorded in the expression patterns acquired using *TUA2*, *PP2A*, and *TUA2+PP2A* compared to two least stable RGs, except when control (3% sucrose) was used as the calibrator for each developmental stage separately.

Regarding gene expression, elevated sucrose levels led to notable upregulation of *Susy1* in later stages, particularly T3 (**Figure 9A**). A similar pattern was observed for *CINV1* in T2, but to a lesser extent (**Figure 9C**). In contrast, 6% sucrose led to downregulation of *SPS1* in T2 and T4 (**Figure 9B**).

## 4 Discussion

Assessment and profiling of gene expression are important and widely applied approaches for understanding the roles of various genes in diverse biological processes. For this purpose, RT-qPCR remains the preferred method due to its speed, high sensitivity and specificity, as well as reproducibility (Quackenbush, 2002; Nolan et al., 2006). However, a prerequisite for accurate interpretations of qPCR results is the use of reliable and stable internal RGs for data normalization using the 2^−ΔΔCt^ method (Livak and Schmittgen, 2001). Inappropriate correction of target gene data can lead to misinterpretation of gene expression results and, consequently, erroneous conclusions.

To date, only actin has been used as a RG for normalizing gene expression data in kohlrabi samples (Ćosić et al., 2019; Ćosić et al., 2021; Nuruzzaman et al., 2022); however, its suitability as an internal reference has not been properly evaluated. Consequently, the lack of accessible information on RGs and reliable standardization of expression data for this important cultivar may considerably affect future studies on gene activity, such as those related to mechanisms underlying carbohydrate assimilation and tuber formation.

Besides *actin*, the most commonly applied RG, several other housekeeping genes such as *18S*, *TUB* and *GAPDH* are frequently used for the normalization of qPCR data, demonstrating consistency and high efficiency across various plant species (Gutierrez et al., 2008; Li et al., 2021; Zhang et al., 2021). Conventionally, RGs are associated with vital metabolic processes, such as phosphorylation (*PP2A*; Zhou et al., 2004), photosynthesis (*GAPB*; Simkin et al., 2023), Ca^2+^ signaling (*GLUR3.2*; Gangwar et al., 2021), gene regulation (*HIS3.3*; Wollmann et al., 2017), and post-translational modifications (*UBC9*; Park et al., 2011), or serve as essential structural components of the cytoskeleton (*ACT7*; Gilliland et al., 2003 and *TUA2*; Kopczak et al., 1992). These genes are generally assumed to exhibit stable and constant activity. Numerous studies have indicated that this is not always the case, reporting variation in the expression levels of traditional RGs across different cell types or under diverse conditions (Volkov et al., 2003; Gutierrez et al., 2008; Wan et al., 2017; Mizzoti et al., 2018). Over the past decade, many new genes have been proposed and validated as potential RGs; however it has also been increasingly recognized that multiple stable RGs can typically be identified for any given set of samples, yet no single gene remains stable across all experimental conditions (Kozera and Rapacz, 2013). This implies substantial variability in the expression stability of potential RGs due to differences in species, growth conditions, developmental stages, tissues and treatments—that is, across different experimental contexts (Jain et al., 2006; Gutierrez et al., 2008; Gao et al., 2012; Zheng et al., 2018).

Earlier studies indicated that using a single RG for the normalization of gene expression data can lead to inaccurate and erroneous results (Vandesompele et al., 2002). This further confirms that the selection and optimization of not just one but several suitable RGs for each experimental setting are necessary for the correct interpretation of qPCR results. Despite this knowledge, many studies still overlook the importance of proper scientific data processing (Ferreira et al., 2023).

In the present study, fifteen candidate RGs were selected for stability evaluation. The majority of these genes have been previously studied as candidate RGs in various plant species (Hu et al., 2009; Wan et al., 2011; Tian et al., 2015; Zhu et al., 2019; Xie et al., 2021; Škiljaica et al., 2022; Ferreira et al., 2023), highlighting the need for optimization of the qPCR data normalization process. The PCR amplification efficiencies of the selected RGs, determined using specifically designed primer pairs, ranged from 92.40% to 101.45%, with corresponding R^2^ values from 0.996 to 1.000, indicating high accuracy and sensitivity. In contrast, the average Cq values of the candidate RGs varied widely, ranging from 4.594 (*18S*) to 28.935 (*F-box*), signifying great variation in RG expression levels across the samples and pointing to the lack of constitutive and stable expression required for reliable internal controls. Comparable wide variation in Cq values among candidate RGs has been reported in other plant species such as *Eucommia ulmoides* (Ye et al., 2018), *Liriodendron chinense* (Tu et al., 2019), *Cryptomeria fortunei* (Zhang et al., 2021), and *Stipagrostis pennata* (Li et al., 2021).

Individual application of the most commonly used statistical algorithms, geNorm, NormFinder, BestKeeper and ∆Ct method, for assessing RG stability can sometimes lead to ambiguous conclusions, due to the discrepancies in the selection of the most stable gene (Tu et al., 2019; Yu et al., 2019; Zhang et al., 2021). This is possibly due to differences in the underlying approaches and mathematical models of each statistical algorithm used. Variations in stability analysis results among the applied algorithms were also observed in our study. For example, for the developmental stage group, *TUA2* and *SAND* showed the highest stability according to both NormFinder and the ∆Ct method, while GeNorm and BestKeeper identified *TIP41* and *18S* as the most stable genes, respectively. Consequently, the online RefFinder tool (Xie et al., 2012; Xie et al., 2023) provides a comprehensive analysis by integrating these four different algorithms, and calculating the geometric mean to generate a final ranking list of the optimal RGs.

None of the analyzed RGs exhibited consistent stability across the different experimental conditions. Although *18S* displayed the least fluctuation in expression levels among all samples, *TUA2*, *ACT7* and *SAND* were suggested as reliable options for investigating gene activity in the kohlrabi system according to the comprehensive analysis, with *TUA2* distinguished as potentially the best choice since it was ranked among the top five RGs on the final ranking list in all tested experimental groups. Additionally, *Ubc12* was often listed among the five most stable genes by each of the four algorithms separately, and was ranked in the top half of each of the ten experimental category-based lists as obtained by RefFinder*. UBC* was one of the genes suggested as a good selection for gene expression analysis in *E. ulmoides* (Ye et al., 2018) and *C. fortunei* (Zhang et al., 2021).

Tubulin family genes are undeniably recognized as reliable RGs across both the plant and animal kingdoms (VanGuilder et al., 2008; Rentoft et al., 2010). They have been established as good internal references for qPCR analysis and have frequently been used in various plant species and their tissues/organs exposed to various experimental setups, including different flower developmental stages of *Arabidopsis thaliana* (Ferreira et al., 2023), *Salix matsudana* (Zhang et al., 2017) under abiotic stress, *Baphicacanthus cusia* (Huang et al., 2017) under hormone stimuli, and *Daucus carota* or *Fragaria vesca* under heat treatment (Tian et al., 2015; Liu et al., 2020). According to the comprehensive ranking in RefFinder, *TUA2* could be the most suitable RG if only a single gene is used as a calibrator in kohlrabi data normalization. Indeed, *TUA2* displayed overall the greatest stability in expression throughout the sample sets; it was ranked in top five RGs in all experimental sets, except the T4. *TUA2* was ranked first for the developmental stage, the sucrose and the T2 stage datasets which was in accordance with the ∆Ct method. However, as for the remaining individual methods, it was ranked first only by NormFinder in the case of the developmental stage and the T2 sets, and first for the sucrose set according to geNorm but not according to BestKeeper. In species other than kohlrabi, some studies pointed to inconsistent activity of these genes when exposed to different conditions. For example, *TUBα* was generally considered the most unstable RG in spinach (Xie et al., 2021). Also, hormone treatments, among others, led to significant variation in *TUB* activity in *Achyranthes bidentata* (Li et al., 2017).

Similar to tubulin, *actin* is a well-known RG, probably even more popular as a reference gene in expression analyses. Various studies included *actin* in qPCR data processing, demonstrating the significance of traditional RGs in specific experimental setups (Tian et al., 2015; Wang et al., 2016; Ni et al., 2019; Zhu et al., 2019). *Actin* was one of the topmost ranked genes for studying *C. fortunei* under different types of abiotic stress and hormone stimuli (Zhang et al., 2021) and also *A. thaliana* reproductive tissues using different mutant lines (Ferreira et al., 2023). Nevertheless, our study indicated that *ACT7* was not suitable for all analyzed groups in the kohlrabi system. For example, it was ranked very low in the developmental stage data set, in accordance with the RefFinder comprehensive evaluation; however, both individual algorithms and the comprehensive ranking identified *ACT7* as the most stable internal control in the CK group, together with individual TDZ treatment.

Regarding *SAND*, a relatively novel RG candidate with a conserved domain and primary function in transcriptional regulation (Bottomley et al., 2001; Cottage et al., 2001), it emerged as a suitable RG in the comprehensive analysis, ranking among the five most stable genes in six out of ten experimental sets, including the total set of samples. All four individual algorithms jointly confirmed *SAND* as the optimal choice only for the developmental stage; moreover, for the majority of experimental sets, at least one of the algorithms ranked *SAND* within the top five. *SAND* was one of the most stable genes across numerous cabbage samples tested, in various types of tissue under different experimental conditions such as hormone treatments, temperature and drought–induced stress (Brulle et al., 2014; Zeng et al., 2020). Optimal application of *SAND* as RG was confirmed under hormone treatment in *Oenanthe javanica* (Jiang et al., 2014) and during biotic stress in the susceptible grapevine cultivar Trincadeira (Monteiro et al., 2013), as well. Furthermore, Yang et al. (2024) demonstrated that this gene exhibited the highest stability in different *Commelina communis* tissues under various abiotic stress conditions, including drought, herbicide and copper treatments.

*18S* is also widely recognized as a universal reference gene, having been extensively used for normalization across a broad range of species in the past studies. It was shown to be optimal RG in *C*. *fortune* (Zhang et al., 2021), *Panicum miliaceum* (Yue et al., 2016), as well as in rice (Jain et al., 2006) and spinach (Xie et al., 2021) during stress treatments. While *18S* showed the most consistent Cq values overall in our study, it underperformed compared to the three aforementioned RGs when evaluated through RefFinder and related algorithms. Underlying reasons for this phenomenon are likely related to high abundance of *18S*, which creates a great difference between Cq values when compared to transcripts of selected target genes, and this affects the normalization process (Bustin et al., 2009). In addition, *18S*, as ribosomal RNA, have different degradation kinetics than mRNA so its stability does not always precisely correspond to the physiological status of the mRNA abundance (Vandesompele et al., 2002; Hellemans et al., 2007). Hence, algorithms like RefFinder may exclude *18S* as reliable RG compared to other, mRNA candidates. However, its stability in our system was stage-specific; in the T1 and T3 groups, *18S* proved highly reliable, ultimately ranking second most stable gene among tested RGs in the comprehensive analysis for these developmental phases. In contrast, low stability of this gene was detected in the course of flower buds development in *Brassica rapa* (Xu et al., 2014).

According to both individual algorithms and RefFinder calculations *GAPB* and *GLUR3.2* were identified as the least stable RGs in the majority of the investigated groups in our study. Interestingly, although GAPB belongs to the glyceraldehyde-3-phosphate dehydrogenase (GAPDH) enzyme family, the expression stability of encoding gene was low in the kohlrabi system, unlike *GAPDH*, a commonly used RG that has demonstrated high stability in many different plants, tissues and organs, as well as under various environmental settings (Iskandar et al., 2004; Tian et al., 2015; Duan et al., 2017; Liang et al., 2018; Jin et al., 2019; Li et al., 2021). Nevertheless, during the last few decades, several RG validation studies have revealed that *GAPDH* is not always an ideal internal standard across different tissues and conditions in certain plants, such as ramie (Yu et al., 2019), carrot (Wang et al., 2016) and switchgrass (Jiang et al., 2014), confirming our findings. For example, *GAPD* exhibited the greatest variation and was ranked poorly under benzothiadiazole, heat, cold, and salt stress treatments in ramie leaves and roots (Yu et al., 2019). In addition, *GLR2* was shown to have relatively high stability in the seedling subgroup, while it was the least stable in other subsets in *A. thaliana* exposed to heat stress (Škiljaica et al., 2022).

Hence, *TUA2*, *ACT7* and *SAND* could individually be selected as the best RGs for the kohlrabi system, assuming that only one gene is sufficient as an internal reference. Nonetheless, various studies have demonstrated that a single RG usually does not meet the requirements for proper qPCR normalization in complex biological systems. For example, in cauliflower, combinations of *ACT7* and *TUA2* with other RGs were proven as the most stable for heat/salt stress and methyl jasmonate treatment, and drought stress, respectively (Lin et al., 2022). Consequently, using more than one RG or a combination of genes is more suitable and necessary to provide accurate data processing for distinct conditions or tissues (Vandesompele et al., 2002; Škiljaica et al., 2022; Zhang et al., 2021; Ferreira et al., 2023; Yang et al., 2024).

The pairwise variation analysis performed by geNorm software was used to establish the optimal number of RGs required for accurate RT-qPCR normalization in kohlrabi experimental sets. The results indicated that the use of the geometric means of the two top-ranked candidate RGs could be sufficient for data normalization in the same way as the geometric mean of three or more genes, since the V_2_/V_3_ values were below the conventional threshold value of 0.15 (Vandesompele et al., 2002). For all three experimental sets selected for validation of expression (developmental stage, T1 and sucrose), the geometric mean of the two highest-ranked genes—based on the comprehensive analysis—was used as the internal reference. In most cases, no significant difference was detected between relative expressions of target genes when normalized by the most stable corresponding RGs, either individually or using their geometric mean, in all subsets of the developmental stage and T1. However, the increasing complexity of the analyzed data set, such as the sucrose set (comprising different developmental stages and sugar treatments), introduced greater variation in expression. This confirms that large datasets originating from multifaceted experimental setups require thorough and comprehensive optimization of qPCR normalization prior to the final interpretation of results.

Moreover, significant differences occurred in the relative expression patterns calculated using the two most stable genes and their geometric mean compared to the two least stable RGs in all three sets. This indicates that choosing an inappropriate internal reference can lead to an erroneous interpretation of experimental outcomes. This observation held true for normalization of expression levels of all three target genes involved in plant carbohydrate metabolism. For example, the activity of *CINV1*, which is primarily involved in the cytosolic cleavage of sucrose into glucose and fructose, was generally either similar to the control or slightly downregulated when normalized using the two most stable genes, *eIF4A-1* and *18S*, and their geometric mean in the T1 set. However, normalization with *GLUR3.2* (one of the two lowest-ranked genes) led to much more pronounced downregulation; in contrast, using the least stable RG, *GAPB*, resulted in an almost twofold upregulation compared to the control.

Similar findings, indicating that no single RG showed a continuous level of expression or outperformed other candidates under separate experimental conditions, have been reported in many different species and experimental systems. In *A. thaliana*, *TIP41* and *UBC21* proved to be the most stable in buds, *UBC21* and *PUX7* in leaves, while *OGIO* and *PUX7* were the most reliable in seedlings under elevated temperatures (Škiljaica et al., 2022). In addition, *RCE1*, *SAC52* and *TUA2* were the top-ranked RGs in different flower developmental stages, while *YLS8*, *HIS3.3* and *ACT7* exhibited the most stable expression for normalization in *A. thaliana* mutant studies (Ferreira et al., 2023). When subjected to various abiotic stresses, spinach plants also showed variation in different RG stability: *EF1α* and *ARF* were suitable for exposure to CdCl_2_, *RPL2* and *actin* for NaHCO_3_, *ARF* and *CYP* for Na_2_CO_3_, and *18S rRNA* and *COX* for NaCl stress (Xie et al., 2021).

## 5 Conclusions

This study is the first systematic analysis for the selection of suitable reference genes for qPCR analysis in young developing kohlrabi under different hormone and sugar stimuli *in vitro*, and to our knowledge, the overall first attempt to select reference genes for qPCR analysis in kohlrabi. We selected fifteen candidate genes, including both conventionally used RGs and relatively new candidates, based on numerous validation studies of expression data normalization. Our analyses indicate that the most stable RGs vary depending on the treatment and/or developmental stage of the kohlrabi plants. Taken together, *TUA2* could be used as a reference gene for evaluating gene expression across all stages and treatments, highlighting the fact that traditional reference genes should not be overlooked. However, a combination of specific reference genes, such as *TUA2* with *SAND* and *TUA2* with *PP2A*, may provide the most reliable normalization for particular experimental conditions.

Moreover, qPCR utilizing carefully chosen and validated reference genes remains a fundamental method for analyzing gene expression in plants and it is crucial to recognize that no single reference gene maintains stability across all experimental conditions. In that respect, our study provides a valuable framework for selecting appropriate reference genes in non-model plant systems, such as kohlrabi.

## Data Availability

The raw data supporting the conclusions of this article will be made available by the authors, without undue reservation.
