# Supplementary material for: Selection and validation of suitable reference genes for qPCR expression analysis in young in vitro grown kohlrabi under cytokinin and sucrose treatments
Source: Front Plant Sci. 2026 Apr 29;17:1820839. doi: 10.3389/fpls.2026.1820839 (PMC13169185; doi:10.3389/fpls.2026.1820839)

Supplementary Material

Selection and validation of suitable reference genes for qPCR expression analysis in young *in vitro* grown kohlrabi under cytokinin and sucrose treatments

**Anđela Tomić, Jelena Milojević, Martin Raspor, Mariana Stanišić, Branka Uzelac, Slavica Ninković, Tatjana Ćosić**

**Supplementary Figure 1.** The specificity of the designed primers for the 15 selected references genes (RGs). (A) PCR gel electrophoresis with a distinct single band of the expected length for each amplified target fragment for each pair of primers. (B) Melting curve analysis for tested RGs, demonstrating the occurrence of a single peak as an evidence of the amplification of a single amplicon.


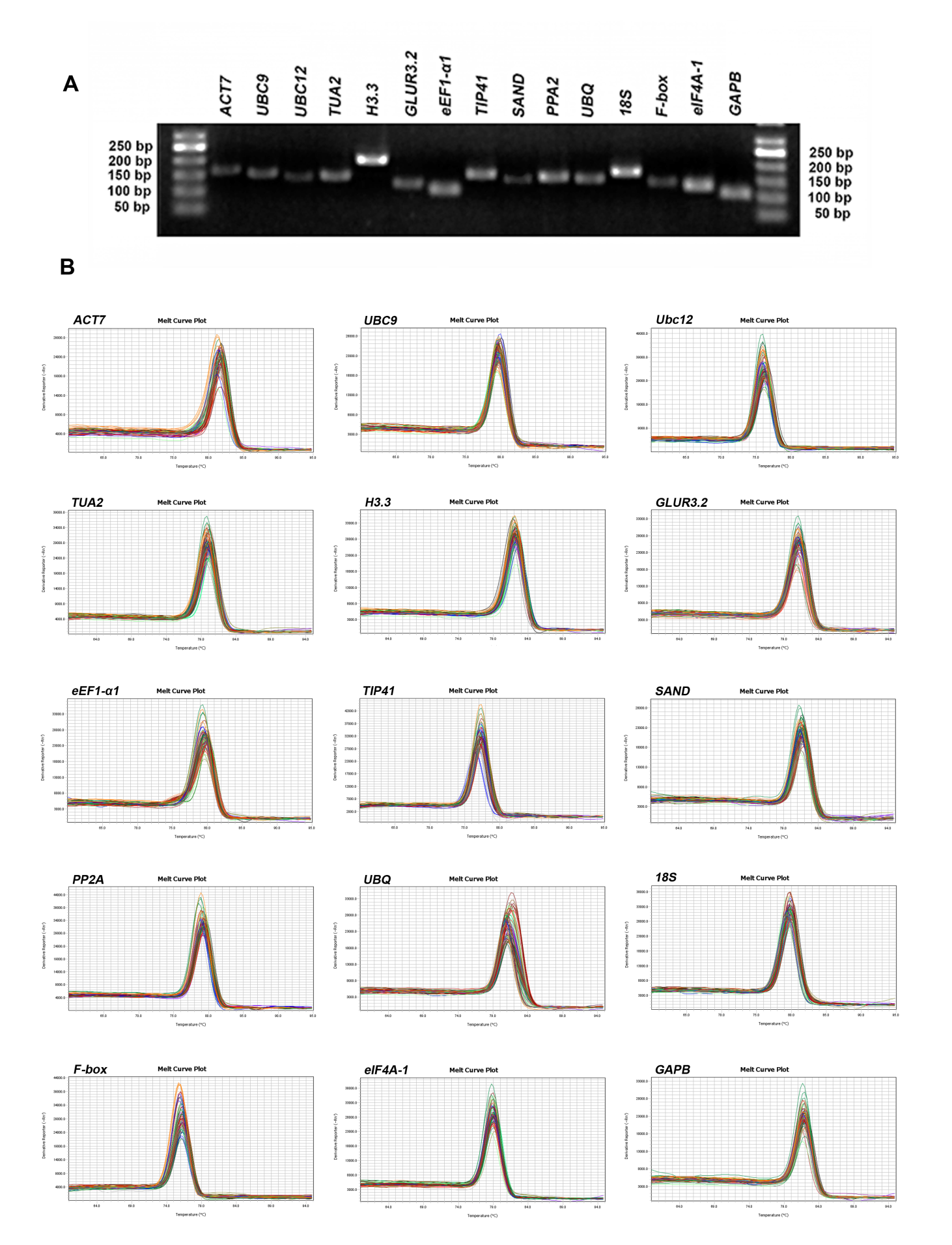

Supplement: Supplementary file 1 [file Table1.docx]
